# Prospective association between the gut microbiota and incident pneumonia: a cohort study of 6419 individuals

**DOI:** 10.1186/s12931-025-03453-w

**Published:** 2025-12-22

**Authors:** Irina Wikki, Joonatan Palmu, Anni Kauko, Aki Havulinna, Pekka Jousilahti, Leo Lahti, Rob Knight, Veikko Salomaa, Teemu Niiranen

**Affiliations:** 1 Department of Internal Medicine, University of Turku, P.O. Box 52, Turku, FI-20521 Finland; 2 Division of Gastroenterology, Turku University Hospital, Turku, Finland; 3 InFLAMES Research Flagship Center, University of Turku, Turku, Finland; 4 Department of Computing, University of Turku, Turku, Finland; 5 Department of Public Health, Finnish Institute for Health and Welfare, Helsinki, Finland; 6 Department of Public Health, Faculty of Medicine, University of Helsinki, Helsinki, Finland; 7 Department of Pediatrics, University of California San Diego, La Jolla, San Diego, CA USA; 8 Department of Computer Science and Engineering, University of California, La Jolla, San Diego, CA USA; 9 Department of Bioengineering, University of California, La Jolla, San Diego, CA USA; 10 Center for Microbiome Innovation, University of California, La Jolla, San Diego, CA USA; 11 Halıcıoğlu Data Science Institute, University of California San Diego, La Jolla, San Diego, CA USA; 12 Division of Internal Medicine, Turku University Hospital, Turku, Finland

**Keywords:** Pneumonia, Butyrate, Incidence, Prospective studies, Microbiota-host interactions

## Abstract

**Background:**

Previous animal studies have identified the protective capacity of the gut microbiota against respiratory infections. Nevertheless, the prospective association between human gut microbiota and pneumonia risk remains unknown.

**Objectives:**

To evaluate the links between gut microbiota and incident pneumonia in a representative population sample.

**Methods:**

We performed shotgun metagenome sequencing on stool samples from 6419 FINRISK 2002 participants. Participants were followed up for incident pneumonia using nationwide health register data. We employed multivariable-adjusted Cox regression models and permutational multivariate analysis of variance (PERMANOVA) to assess the association of gut microbiome alpha diversity, compositional variation (beta diversity), and taxonomic composition with pneumonia risk.

**Results:**

Altogether, 685 patients (10.7%) developed pneumonia during a mean follow-up of 17.8 years. Alpha diversity was not associated with incident pneumonia (hazard ratio [HR] 1.00; 95% confidence interval [CI] 0.93 − 1.08), whereas community composition was (PERMANOVA R^2^ = 0.03%; *P* = 0.02). We observed an inverse association between the relative abundance of butyrate-producing bacteria and incident pneumonia (HR per 1-SD increase 0.91; 95% CI 0.85–0.98). The relative abundance of *Bacteroides_F pectinophilus*,* Eubacterium_G ventriosum*, *Agathobaculum butyriciproducens*,* Butyribacter intestini*,* Eubacterium_I ramulus*,* CAG-1427 sp000435675*,* and CAG-603 sp900066105* were inversely associated with pneumonia risk. The relative abundance of *Clostridium_AQ* innocuum was positively correlated with pneumonia risk.

**Conclusions:**

The gut microbiota composition, and especially the relative abundance of butyrate-producing bacteria, was associated with lower pneumonia risk in the population. These findings warrant further studies to investigate whether microbiome modulation to increase short chain fatty acid production through diet, prebiotics, or probiotics could reduce pneumonia risk.

**Supplementary Information:**

The online version contains supplementary material available at 10.1186/s12931-025-03453-w.

## Introduction

Growing evidence highlights the role of the gut microbiota in maintaining health [1, 2]. Past research has confirmed profound connections between the gut microbiota and distant organs, including connections to the brain, the lungs, the bones, and the liver [3]. The pathophysiology of many diseases is influenced by a complex interplay of the gut microbiota, its metabolites, and the host immune system [4]. Research indicates that the gut-lung axis — a communication pathway between the gut and the lung — is essential for maintaining respiratory health [5]. Disturbances in this axis, influenced by changes in the intestinal microbiota, may facilitate the initiation and progression of pneumonia through immune dysregulation, inflammation, and altered respiratory microbiome [6, 7].

The limited research on the links between the gut microbiota and pneumonia has focused on gut microbiota changes during infection, the association between the gut microbiota and immune responses, and the effect of antibiotics on host defense and disease severity [7–9]. Evidence from these studies suggests that commensal gut microbiota might contribute to the pathogenesis and prognosis of pneumonia. Our recent large, multinational, prospective cohort study demonstrated that the gut microbiota composition, specifically colonization with butyrate-producing bacteria, is related to protection against severe infections [10]. Apart from this single study, prior research has been limited to cross-sectional studies rather than prospective studies that would provide a higher level of evidence. A prospective setting would grant clarity of temporal sequence (did the exposure precede the outcome?) as the findings would not be biased by the disease or its therapy. Furthermore, many of the suggested hypotheses on links between the gut microbiota and pneumonia are based on animal models, and the generalizability of these results to humans remains open due to the considerable differences in animal and human microbiota [11–13].

The main goal of our study was to assess the long-term prospective associations between gut microbiota composition and incident pneumonia, with a special focus on butyrate-producing bacteria. To achieve this goal, we used shotgun metagenomic sequencing to characterize the composition of the gut microbiota in 6419 participants of the FINRISK population survey in 2002. Using nationwide health register data, we then followed these individuals for up to 18 years for hospital-diagnosed incident pneumonia to elucidate the links between the gut microbiota and pneumonia risk.

## Methods

### Study sample

The Finnish Institute for Health and Welfare has conducted FINRISK population surveys at 5-year intervals since 1972 to monitor risk factors for common diseases in the Finnish adult population [14]. For the FINRISK 2002 survey, a stratified random population sample of 13,437 individuals, aged 25–74 years from 6 geographic regions, was drawn from the National Population Information System [14]. The sampling was stratified by sex, region, and 10-year age group, according to the WHO MONICA protocol [15]. Willing participants were asked to provide a stool sample. In total, 8799 individuals participated in the baseline examination and metagenomic sequencing was successfully performed on 7231 stool samples. Participants with incomplete data, with fewer than 50,000 sample reads, or who were pregnant at baseline were excluded from the study. The final study sample consisted of 6419 individuals.

### Stool sampling and metagenomic sequencing

The baseline examination protocol and stool sampling procedure have been detailed in previous publications and summarized in the Online Data Supplement [14, 16]. Metagenomic sequencing was performed at the University of California, San Diego, California. The metagenomic sequencing and stool processing procedures have been described previously [16, 17]. The original sequence data were classified and assigned into microbial taxa using the GreenGenes2 reference database [18].

### Outcome and covariate definitions

Participant data were linked to nationwide health registers for prevalent diseases and incident pneumonia (Table E1). The main outcome was a diagnosis for pneumonia during the follow-up period. This incident event was defined as ICD-10 codes J10.0, J11.0, J12-J16, J17.0, J17.1–8, J18, B01.2, B06.8, and B25.0 recorded in the Hospital Discharge or Causes of Death registers. Diagnoses from both inpatient and outpatient hospital visits are included in the Hospital Discharge Register.

Documented covariates included comorbidities, age, sex, body mass index (BMI), smoking status, alcohol consumption, physical activity, and prior antibiotic use (4 months prior to sample collection). The definitions of these covariates are reported in detail in the Online Data Supplement.

As a sensitivity analysis, we included a Healthy Food Choices score as a covariate in the model to account for the confounding effects of diet [19]. These dietary data were available for 5906 individuals with 591 pneumonia cases.

### Statistical analysis

We used unadjusted and multivariable-adjusted Cox regression models to assess associations between outcomes and continuous microbiota features (alpha diversity, principal components, relative abundance of butyrate-producing and other bacteria, and microbial risk score). We also performed a permutational multivariate analysis (Bray–Curtis dissimilarity at species level, vegan R package, adonis2 function, 999 permutations) to assess the association between community composition and incident pneumonia. We adjusted all multivariable models for age, sex, BMI, alcohol use, smoking, physical activity, prior antibiotic exposure, and comorbidities (diabetes, cardiovascular diseases, cancer, hypertension, pulmonary diseases, and gastrointestinal diseases). We used Kaplan-Meier curves to visualize the temporal associations between microbiota features and incident pneumonia. We defined statistical significance as *P* < 0.05. We applied false discovery rate (FDR) corrected *P* values (Benjamini-Hochberg method) for genus- and species-level analysis. All statistical analyses were performed with R version 4.3.1. The statistical analyses are described in more detail in the Online Data Supplement.

Analysis source codes are openly available at DOI:10.5281/zenodo.13880009.

## Results

The mean follow-up time was 17.8 years, and 685 participants were diagnosed with incident pneumonia. The detailed characteristics of the study sample are reported in Table 1.

**Table 1:** Characteristics of the study sample

		Incident pneumonia	
Characteristic	All (*n* = 6419)	Yes (*n* = 685)	No (*n* = 5734)
Age, median (IQR), y	50.1 (39.0, 59.0)	60.3 (51.1, 67.2)	48.8 (38.1, 57.8)
Follow-up time, median (IQR), y	17.8 (17.7, 17.9)	11.5 (7.7, 14.9)	17.8 (17.8, 17.9)
Men, n (%)	2947 (45.9)	396 (57.8)	2551 (44.5)
BMI, median (IQR), kg/m^2^	26.4 (23.7, 29.4)	27.5 (24.4, 30.5)	26.3 (23.6, 29.2)
Smoking status, n (%)			
Non-smoker	3434 (53.5)	286 (41.8)	3148 (54.9)
Current smoker	1525 (23.8)	183 (26.7)	1342 (23.4)
Ex-smoker	1460 (22.7)	216 (31.5)	1244 (21.7)
Median alcohol usage (IQR), g	36.0 (9.0, 105.9)	30.6 (2.7, 97.5)	38.7 (9.0, 107.6)
Recent antibiotic exposure, n (%)	826 (12.9)	108 (15.8)	718 (12.5)
Physical activity intensity, n (%)			
Light	1353 (21.1)	167 (24.4)	1186 (20.7)
Moderate	3595 (56.0)	410 (59.9)	3185 (55.5)
High	1471 (22.9)	108 (15.8)	1363 (23.8)
Comorbidities, n (%)			
Hypertension	3073 (47.9)	442 (64.5)	2631 (45.9)
Diabetes	352 (5.5)	83 (12.1)	269 (4.7)
CVD	235 (3.7)	65 (9.5)	170 (3.0)
Pulmonary disease	539 (8.4)	114 (16.6)	425 (7.4)
Gastrointestinal disease	102 (1.6)	18 (2.6)	84 (1.5)
Cancer	238 (3.6)	59 (8.4)	179 (3.1)

*Abbreviations*: *IQR* Interquartile range, *SD* standard deviation, *BMI* body mass index, *CVD* cardiovascular disease

The analysis of gut microbiota composition identified 5964 species within 3352 genera. The common taxa that were included in the taxa-level analyses consisted of 384 species and 253 genera.

We first studied the association between alpha diversity (continuous Shannon index) and incident pneumonia using Cox proportional hazards models. Alpha diversity was not significantly associated with incident pneumonia in the unadjusted (hazard ratio [HR] 0.98; 95% confidence interval [CI] 0.91–1.06; *P* = 0.68) or multivariable-adjusted (HR 1.00; 95% CI 0.93–1.08; *P* = 0.95) models.

**Fig. 1:**
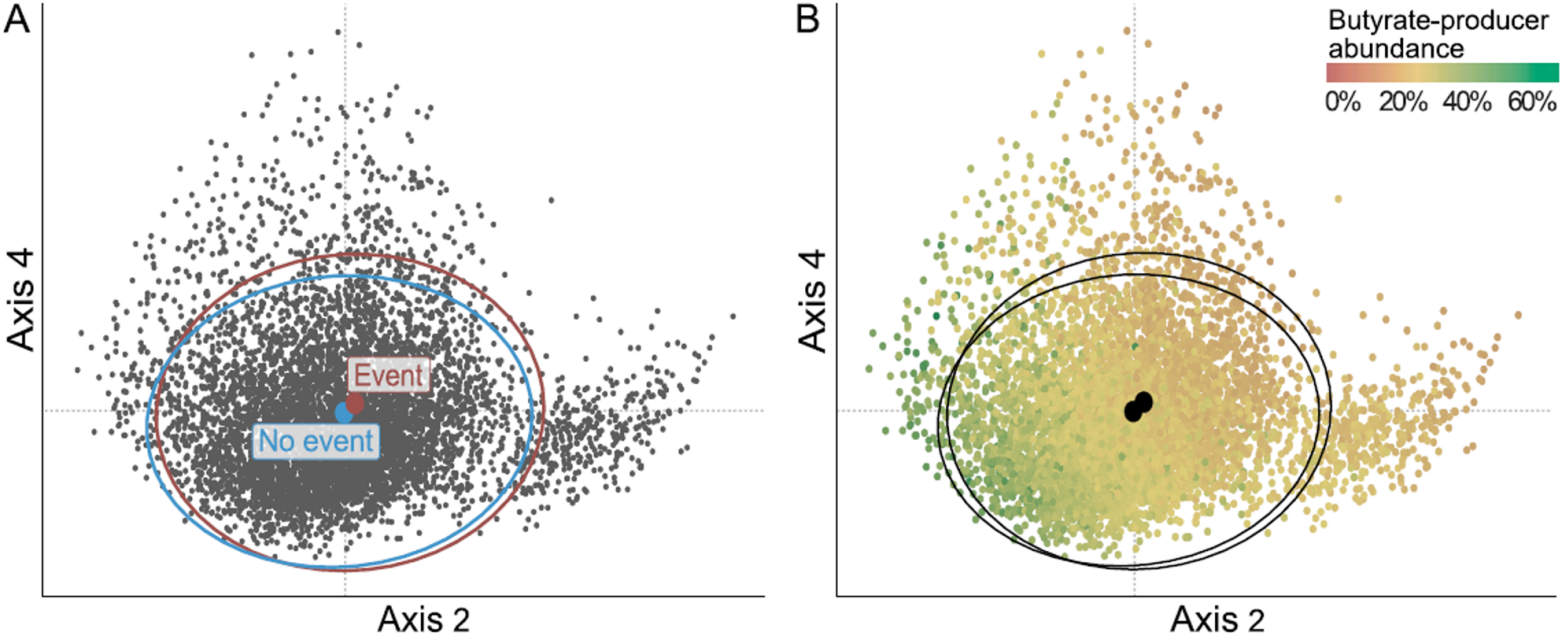
Beta diversity by pneumonia incidence (**A**) and by the abundance of butyrate-producing bacteria (**B)**. **A** PCoA 2 (horizontal axis) and PCoA 4 (vertical axis) are presented in the graph as the most significant axes in multivariable-adjusted beta diversity analysis. **B** Each dot represents one participant, and the color gradient visualizes the total relative butyrate producer abundance of each sample. Circles showing the centroids of our two main groups (event/no event). PCoA = Principal coordinate axis

**Fig. 2:**
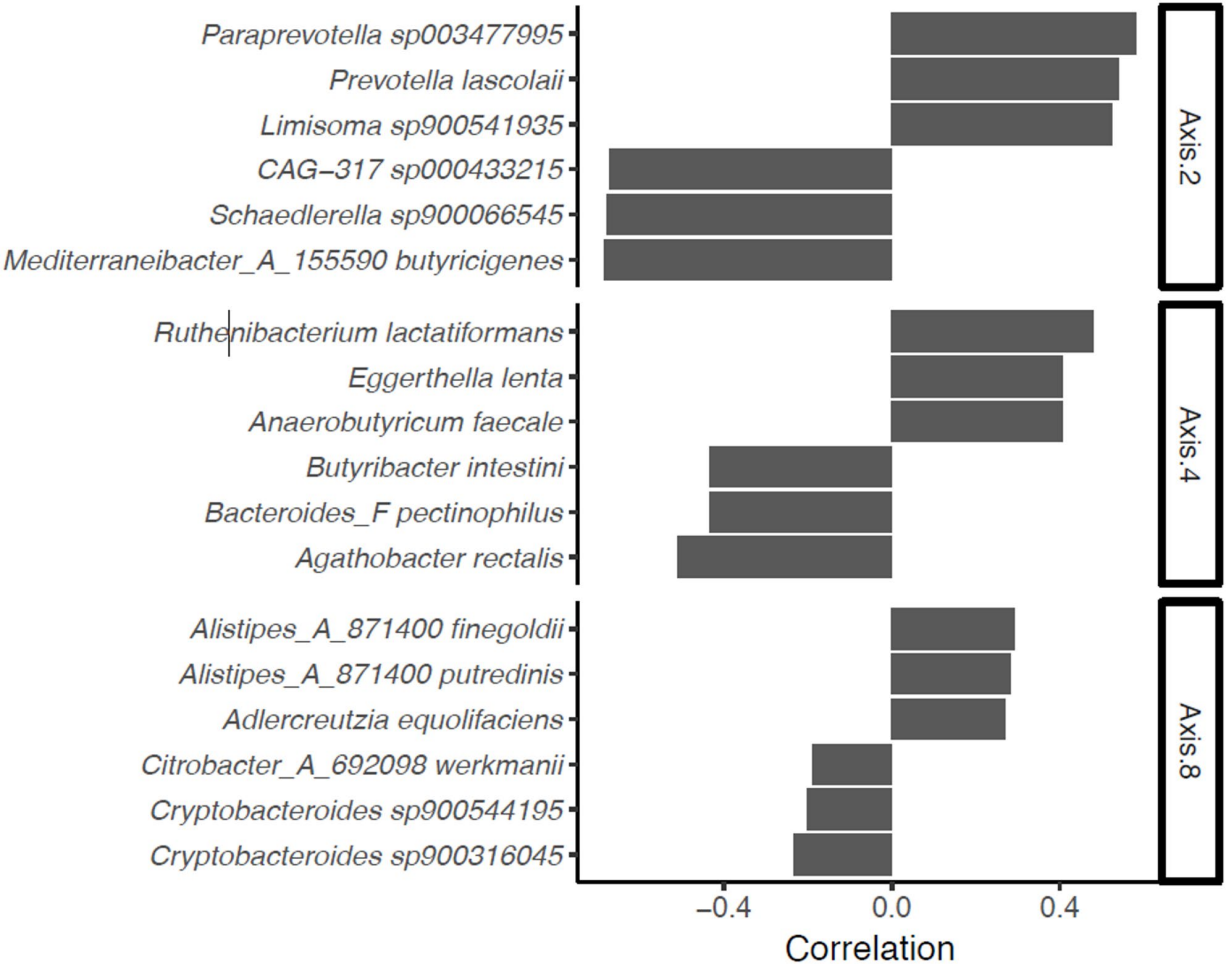
Bacterial species most strongly correlated with PCoA axes 2, 4, and 8. Correlations are reported as Spearman correlations

We then examined the association between gut microbiota community composition and incident pneumonia using the Bray-Curtis dissimilarity indexes calculated at species level, and the first 10 principal coordinates analysis (PCoA) axes. In unadjusted analyses, PCoA axes 8, 4, and 2 were associated with incident pneumonia (*P* ≤ 0.01 for all; Fig. 1). Figure 2 shows the bacterial species that were the strongest correlates of these axes. After multivariable-adjustment, axis 4 remained significant (HR 1.14 per 1-SD increment; 95% CI 1.06–1.22; *P* = 0.005). We also performed permutational multivariate analysis of variance (PERMANOVA) to analyze the association of community composition and incident pneumonia. Both unadjusted (R^2^ = 0.05%; *P* = 0.001) and multivariable-adjusted (R^2^ = 0.03%; *P* = 0.02) analyses demonstrated a correlation between community composition and pneumonia risk.

We employed Cox models to further examine bacterial community composition and identify signature taxa linked to pneumonia risk. We observed 63 common genera significantly correlated with incident pneumonia in the unadjusted genus-level analyses (FDR < 0.05, Table E3). In the multivariable-adjusted models, *Bacteroides F*,* Eubacterium G*,* CAG-603*,* Clostridium AQ* and *Slackia A* remained significantly associated with pneumonia (Table 2). In unadjusted species-level analyses, we observed 100 common species with a significant association with incident pneumonia (FDR < 0.05, Table E4). Eight species were significantly associated in multivariable analysis: *Bacteroides_F pectinophilus*, *Eubacterium_G ventriosum*, *Agathobaculum butyriciproducens*,* Butyribacter intestini*,* Eubacterium_I ramulus*,* CAG-1427 sp000435675*,* CAG-603 sp900066105*, and *Clostridium_AQ* innocuum (Table 2). When the Healthy Food Choices score was included among the covariates, only *Eubacterium_G ventriosum* (HR 0.85; 95% CI 0.79–0.92; *P* = 0.03) remained significantly associated with pneumonia in the species-level analysis, while the genus-level analysis did not yield any significant results (Table E5). As the point estimates remained relatively similar compared to the main results after adjusting for diet, this decrease in the number of significant taxa was mainly a result of decreased statistical power arising from lower number of participants with dietary data available.

**Table 2:** Gut microbial taxa significantly associated with incident pneumonia using Cox proportional hazards model

Taxa	HR	95% CI	*P*	FDR
Genus				
*Eubacterium_G*	0.86	0.80–0.93	7.7 × 10^− 5^	0.02
*Bacteroides_F*	0.87	0.81–0.94	7.9 × 10^− 4^	0.02
*CAG_603*	0.89	0.83–0.95	8.6 × 10^− 4^	0.04
*Clostridium_AQ*	1.13	1.06–1.22	3.8 × 10^− 4^	0.03
*Slackia_A*	1.14	1.06–1.23	7.9 × 10^− 4^	0.04
Species				
*Eubacterium_G ventriosum*	0.84	0.78–0.91	7.1 × 10^− 6^	0.003
*Agathobaculum butyriciproducens*	0.87	0.80–0.93	1.7 × 10^− 4^	0.03
*CAG-1427 sp000435675*	0.87	0.81–0.94	7.6 × 10^− 4^	0.04
*Bacteroides_F pectinophilus*	0.87	0.81–0.94	3.7 × 10^− 4^	0.04
*Butyribacter intestini*	0.88	0.81–0.94	5.2 × 10^− 4^	0.04
*Eubacterium_I ramulus*	0.88	0.81–0.95	9.9 × 10^− 4^	0.05
*CAG-603 sp900066105*	0.89	0.82–0.95	7.4 × 10^− 4^	0.04
*Clostridium_AQ innocuum*	1.13	1.06–1.21	5.1 × 10^− 4^	0.04

Hazard ratios are reported per 1 SD increment

*Abbreviations*: *HR* Hazard ratio, *CI* Confident interval, *FDR* False discovery rate corrected *P*

**Fig. 3:**
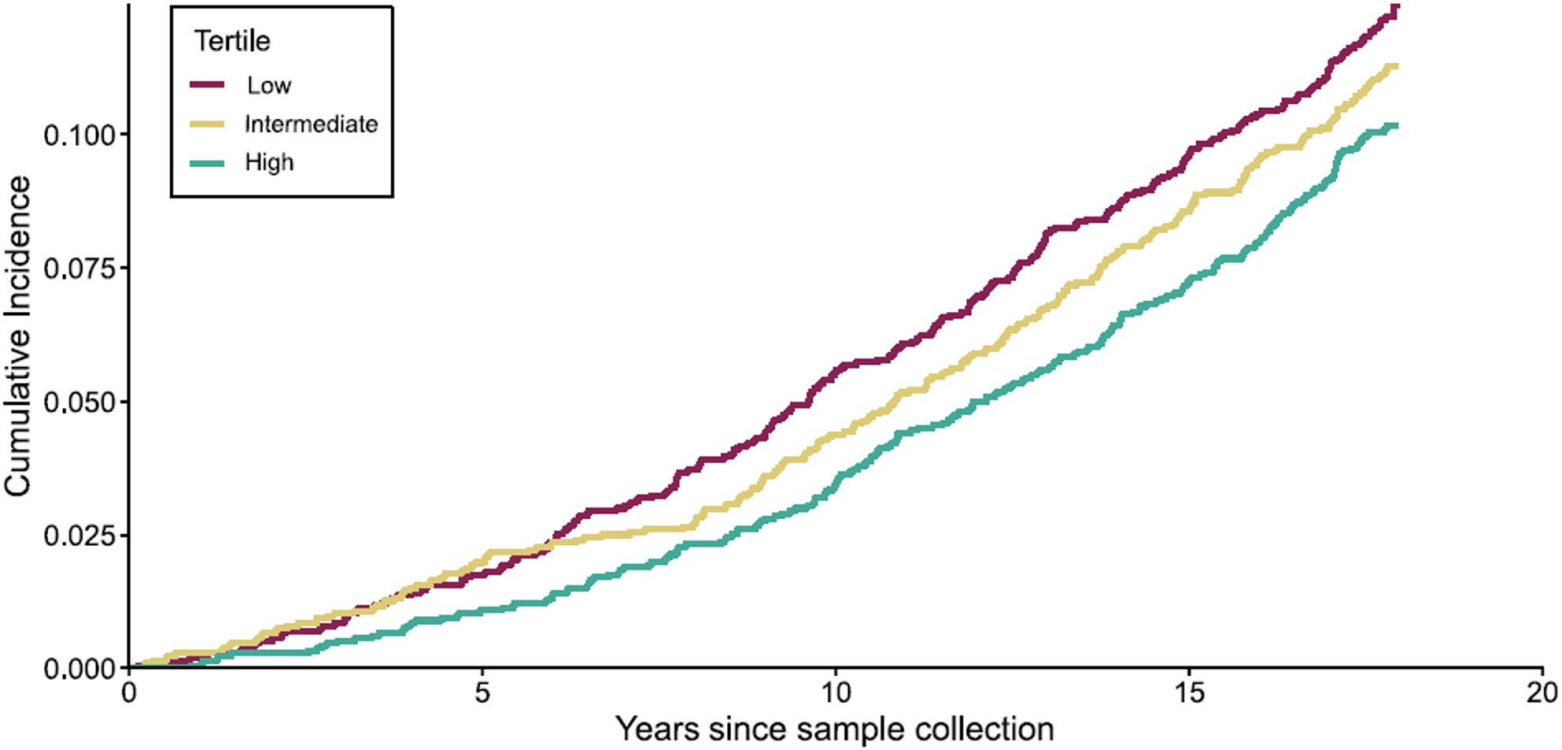
The association of butyrate-producing bacteria abundance with pneumonia incidence. Participants were divided into subgroups based on the abundance of butyrate producers. The plot shows the cumulative pneumonia incidence in subgroups of low, intermediate, and high butyrate abundance

Our next objective was to determine whether the relative abundance of butyrate-producing bacteria is associated with pneumonia risk. We observed an inverse association between butyrate-producing bacteria relative abundance and pneumonia incidence in unadjusted analyses (HR per 1-SD increase 0.90; 95% CI 0.84–0.97; *P* = 0.005; Fig. 3; Table 3). This significant association was robust to adjustment for covariates (HR per 1-SD increase 0.91; 95% CI 0.85–0.98; *P* = 0.01).

**Table 3:** The association of butyrate-producing bacteria abundance with pneumonia incidence

	Unadjusted		Adjusted	
Butyrate producer abundance	HR (95% CI)	*P*	HR (95% CI)	*P*
Continuous	0.90 (0.84–0.97)	0.005	0.91 (0.85–0.98)	0.01
Tertiles				
Low	1.00 (ref)		1.00 (ref)	
Intermediate	0.91 (0.76–0.1.09)	0.30	0.93 (0.77–1.11)	0.41
High	0.81 (0.67–0.97)	0.02	0.79 (0.66–0.95)	0.01

Hazard ratios are reported per 1 SD increment

*Abbreviations*: *HR* Hazard ratio, *CI* Confident interval, *FDR* False discovery rate corrected *P*

Finally, we created a bacterial risk score for incident pneumonia using elastic nets for Cox models (see Online Data Supplement). A detailed portrayal of individual species-level taxa contribution to the risk score is reported in Figure E1 and Table E6. The risk score was calculated for each individual, and the cohort was divided into tertiles based on the risk score. As seen in Figure E2, the multivariable-adjusted incidence of pneumonia was higher in the high-risk score group (HR 1.31; 95% CI 1.08–1.59; *P* = 0.007) when compared to the low-risk group. A 1-SD increment in the risk score resulted in a 1.07-fold increase (95% CI 1.02–1.13; *P* = 0.008) in pneumonia risk. As a sensitivity measure, we successfully validated the risk score by dividing the cohort population into two equal-sized subpopulations (see Online Data Supplement).

## Discussion

In this prospective cohort study, we investigated for the first time the association between gut microbiota composition and long-term pneumonia incidence, using secondary health care diagnoses from a representative population sample. We demonstrated that specific axes of microbiota community composition were associated with pneumonia risk. In addition, a higher relative abundance of butyrate-producing bacteria in the gut microbiota was associated with a lower risk of incident pneumonia.

We found no correlation between alpha diversity of gut microbiota and pneumonia risk. This is in contrast to prior cross-sectional studies, which have mainly reported a loss of alpha diversity in infected patients when compared to healthy controls [20]. One of these studies suggested that the extent of alpha diversity loss may also be related to the severity of the disease [21]. However, evidence from cross-sectional and prospective studies are not directly comparable because the infection and its treatment may alter the composition of the gut microbiota. Our findings are corroborated by some of the earlier literature. Orieux et al. demonstrated that the difference between critically ill patients who later developed pneumonia and those who did not develop pneumonia was linked to differences in the relative abundances of gut microbial taxa rather than alpha diversity [22]. Li et al. also presented similar findings in a study on stroke-associated pneumonia [23]. Interestingly, Maji et al. reported an increased alpha diversity and elevated abundance of bacteria that produce butyrate and other short-chain fatty acids (SCFAs) in patients with active pulmonary tuberculosis infection [24]. These prior findings, along with our results, may suggest that the functional capacity of the gut microbiota may be more important than bacterial diversity in maintaining intestinal balance.

Data on the causal relationship between gut microbiota and lung infections are scarce and mainly based on experimental animal studies. In a small prospective study of critically ill patients, dysbiotic gut microbiota were found to be associated with increased risk of infection, accompanied by impaired immune system function [25]. Most evidence focuses on the behavior of gut microbiota during infection. Mouse studies have shown notable shifts in intestinal microbial communities during bacterial and viral lung infections, including loss of alpha diversity and altered community composition [26, 27]. Additional data from mice suggest that the gut microbial community boosts the immune system and supports recovery from bacterial pneumonia [9, 26]. Some studies indicate that the observed changes in gut microbiota composition during infection result from the infection rather than influence susceptibility to it. Nevertheless, a growing body of research from various areas, such as lung diseases, suggests that changes in the gut microbiota may precede disease manifestation [28, 29].

Differential taxa analysis showed a positive association of two common genera and one common species with incident pneumonia. *Clostridium_AQ innocuum* has been identified as a common inhabitant of the human gut and has been linked to gastrointestinal diseases [30]. It has also been identified as a rare cause of bacteremia and endocarditis. A recent study of this bacterial species suggested it may be less benign in the gut environment than previously considered [31]. The other observed genus with positive association with pneumonia, *Slackia_A*, has previously been positively linked to various disease states such as pulmonary artery hypertension, type 2 diabetes, and colorectal cancer [32, 33]. These previous findings provide some support for our results, although the mechanism of how colonization with these bacteria may increase the risk of pneumonia remains unclear.

We observed seven species that were inversely associated with incident pneumonia. *Agathobaculum butyriciproducens* has recently been identified as a strictly anaerobic bacteria that produces butyric acid [34]. *Eubacterium_I ramulus* has been identified as a gut commensal metabolizing dietary flavonoids that may mediate antioxidative, anti-inflammatory and antimicrobial effects [35–37]. *CAG-1427 sp000435675* has not, to our knowledge, been studied, but belongs to the phylum *Actinobacteriota* that is referred to as “a relevant minority for the maintenance of gut homeostasis” [38]. *Bacteroides_F pectinophilus*,* Butyribacter intestini*,* CAG-603 sp900066105*, and *Eubacterium_G ventriosum* belong to the family *Lachnospiraceae*, which are commensal anaerobic bacteria and are among the main SCFA producers in the gut. The *Lachnospiraceae* family of bacteria have been identified as enhancing the immunological function in the gut, although their impact on the host physiology has been inconsistent across different studies [39]. Many observational studies have reported a depletion of *Lachnospiraceae* family of bacteria during respiratory infections [20]. *Eubacterium_G ventriosum* is recognized as one of the key butyrate-producing bacteria in the gut (Table E2). A small observational study has reported a significant depletion of *Eubacterium ventriosum* in both COVID-19 patients and COVID-negative pneumonia patients, when compared to healthy controls [40]. In COVID-19 pneumonia patients, a significant inverse correlation between SCFA-producing bacteria and inflammatory markers was observed [41]. Studies on kidney transplant recipients and patients post-allogenic HSCT have reported that reduced SCFA-producing bacteria are correlated with increased risk and incidence of viral respiratory tract infections [42, 43]. The results of our species-level analyzes and the results from previous studies therefore highlight the importance of butyrate and other SCFA-producing bacteria as a potential modifier of infection risk.

Our results on butyrate-producing bacteria are in agreement with outcomes from previous cross-sectional studies. Abundant evidence from earlier studies has demonstrated the immunomodulatory and health-promoting potential of gut-derived SCFAs, especially butyrate, acetate and propionate. The proposed mechanisms underlying these findings include direct and indirect bi-directional signaling pathways between the gut microbiota and distant organs. Intraluminal production of butyrate and other SCFAs is known to contribute to the stability of the intestinal microenvironment by supporting the growth of commensal bacteria and the integrity of the intestinal barrier [44, 45]. Butyrate and other SCFAs are also shown to possess antimicrobial properties that inhibit the growth of various Gram-positive and Gram-negative bacteria [45, 46], including many opportunistic pathogens. Evidence from in vitro and in vivo mouse studies suggests that butyrate enhances the antibacterial clearance of macrophages, providing protection both locally in the intestine and distant organs such as the lungs [47]. Acetate has also been shown to increase the bactericidal activity of macrophages during pneumonia [48]. Furthermore, butyrate and other SCFAs stimulate and support the development of helper-T cells, which play a role in immune regulation and neutrophil recruitment within the lungs [49, 50]. Another study demonstrated that SCFAs increase the number of activated macrophages and reduce neutrophil-attracting chemokine CXCL1 production in the airways, modulating neutrophil recruitment and thus reducing tissue damage [51]. Moreover, butyrate has been demonstrated to modulate dendritic cell production of inflammatory cytokines, which is significant particularly in the later stages of infection [47, 52]. The depletion of SCFA-producing bacteria has been linked to impaired pulmonary defense against bacterial pathogens [53], and especially acetate supplementation has resulted in reduced bacterial loads in the lungs [48]. Several other studies have also confirmed a positive association between gut SCFA production and its immunomodulatory capacity [54, 55]. These previous findings deepen our understanding of the mechanisms underlying the potential protective capacity of SCFA-producing bacteria against pneumonia.

The taxa used for the calculation of the abundance of butyrate-producing bacteria (Table E2) do not only produce butyrate, but many are also producers of other SCFAs, including propionate and acetate. Commensal gut bacteria present cross-feeding properties, where the diversity and quantity of SCFA production varies according to available substrates and present species [55]. As both acetate and propionate may enter the systemic circulation in greater amounts than butyrate, it may be argued that they have a more potent systemic effect than butyrate. Thus, all major SCFAs, including butyrate, may have a role in pneumonia defense. It should also be noted that we did not perform direct measurement of butyrate or other bacterial metabolites. Therefore, our conclusions are somewhat speculative and based on previous literature and on the assumption that the abundance of butyrate-producing bacteria reflects the intraluminal abundance of butyrate. Further research with direct measurement of intestinal butyrate levels is necessary to validate our findings.

We identified a selection of bacterial taxa associated with pneumonia incidence, and developed a cumulative risk score for an overall risk assessment. Modulation of the gut microbiota has been shown to be effective in treatment recurrent *Clostridioides difficile* infections and is proposed as a potential treatment for many other dysbiosis-associated diseases [56]. By analyzing the composition of gut microbiota, it could be possible to find patients at high risk of future infections. The abundance of SCFA-producing bacteria could potentially be considered as a marker for infection risk given the aforementioned and other established benefits of butyrate and other SCFAs [52, 57]. Our risk score presents one useful way of examining the composition of the gut microbiota and identifying specific bacterial taxa that are positively or negatively associated with the risk of infection. Such a risk score might also be used to identify individuals who would benefit from prophylactic gut microbiota modulation. However, further experimental research is needed to confirm the benefit and safety of such a prophylactic approach. Furthermore, it remains unclear to what extent our risk score is applicable at a general level, as it has not been externally validated.

The strengths of our study include a representative population sample with comprehensive nationwide register-based follow-up with virtually no loss to follow-up. The primary strength is that this is the first report on the long-term prospective association between gut microbiota and incident pneumonia, thus avoiding many biases inherent in cross-sectional studies [58]. However, our study focused on more severe forms of pneumonia that required hospital evaluation or hospitalization. Neither was there detailed information on the type of pneumonia (i.e., viral vs. bacterial infection, or community- vs. hospital-acquired). This limitation may confound the observed associations, as biological mechanisms for and susceptibility to different pneumonia types vary. Although the Finnish health registers are generally considered highly reliable for research use [59], errors or omissions in the coding process may still occur. Additionally, despite the relative stability of the intestinal core microbiota over time [60], a number of variables could alter it between the time of sampling and the endpoint event. These variables include exposure to long- or short-term medications, such as antibiotic treatments, dietary changes, alterations in environmental factors, and prevalent disease status. In our study, gut microbiota was assessed only at baseline, which may lead to underestimation of the association between the microbiota and pneumonia risk. Repeated stool sampling could provide a more dynamic assessment of an individual’s gut microbiota. As with all observational studies, the associations do not prove causality even in a prospective setting. The presence of unknown or unmeasured confounding factors cannot be excluded and therefore residual confounding may persist.

This research represents the first steps toward a more profound understanding of the links between gut microbiota and pneumonia risk. In short, we demonstrated that the abundance of butyrate-producing bacteria is associated with a lower long-term risk of pneumonia. We identified seven species associated with reduced pneumonia incidence, many of them linked to SCFA production in the gut, and detected a signature of gut microbiota predicting the risk of incident pneumonia. Future implications include the potential for assessing individual infection risk and developing novel treatments for patients in the high-risk group. Randomized clinical trials in patients at high risk of pneumonia that focus on increasing gut SCFA levels are now needed to confirm causality.

## Supplementary Information

Supplementary Material 1.

## Data Availability

The metagenomic data are available from the European Genome-Phenome Archive, (https://ega-archive.org/studies/EGAS00001005020) (accession number EGAD00001007035). The phenotype data contain sensitive information from healthcare registers and they are available through the THL biobank upon submission of a research plan and signing a data transfer agreement (https://thl.fi/en/research-and-development/thl-biobank/for-researchers/application-process).
