# Supplementary material for: Prospective association between the gut microbiota and incident pneumonia: a cohort study of 6419 individuals
Source: Respir Res. 2025 Dec 22;26:354. doi: 10.1186/s12931-025-03453-w (PMC12751896; doi:10.1186/s12931-025-03453-w)
Supplement: Supplementary file 1 — Supplementary Material 1. [file 12931_2025_3453_MOESM1_ESM.docx]

**THE PROSPECTIVE ASSOCIATION BETWEEN GUT MICROBIOTA AND INCIDENT PNEUMONIA: A COHORT STUDY OF 6419 INDIVIDUALS**

Irina Wikki, Joonatan Palmu, Anni Kauko, Aki Havulinna, Pekka Jousilahti, Leo Lahti, Rob Knight, Veikko Salomaa, Teemu Niiranen

ONLINE DATA SUPPLEMENT

**Supplementary Methods**

*Baseline examination*

The participants of FINRISK 2002 were sent an invitation letter with a detailed health questionnaire to fill in at home. The questionnaire included sections on medical history, medications, lifestyle, nutritional, sociodemographic, and psychosocial factors. Participants underwent a standardized physical examination and blood sampling by trained nurses at local health centers or other study sites. The baseline examinations took place in January-March 2002.

*Fecal sampling and data filtering*

All willing participants were asked to collect a stool sample at home and given a pre-paid stool sample kit including a Falcon tube and sampling instructions. The stool samples were mailed overnight between Monday and Thursday under Finnish winter conditions to the laboratory of the Finnish Institute for Health and Welfare. They were frozen as such at -20C and held unthawed until 2017 when metagenomic sequencing was performed. Samples with fewer than 50000 reads were excluded from further analysis.

*Register linkage for prevalent diseases and incident infections*

All permanent residents in Finland are assigned a unique personal identity number at birth or after immigration, ensuring reliable linkage to electronic health registers. Data from patient visits in healthcare units are collected to national healthcare registers maintained by the Finnish Institute for Health and Welfare. These health registers ensure in practice 100% coverage of all major health events (Hospital Discharge Register) and prescription drugs (Drug Purchase and Reimbursement Register). The Causes of Death Register is maintained by Statistics Finland. The quality of the diagnoses in the Finnish national registers has been previously validated (1, 2).

*Outcome and covariate definitions*

We defined body mass index as nurse-measured weight (kg)/height (m)^2^. Smoking status was defined as a non-smoker, ex-smoker, or current smoker. Alcohol use was reported as average weekly alcohol consumption in grams within the last year. Physical activity was categorized as light activity (eg. household tasks or watching TV), moderate activity (at least 4 hours per week non-conditioning activities eg. walking, fishing or hunting), or high activity (regular conditioning activities at least 3 hours per week eg. running, skiing, swimming, or competitive-wise exercise). Commuting or work-related physical activities were excluded. Smoking status, alcohol usage and physical activity were self-reported.

The healthy food choices (HFC) score was included as a covariate in a sensitivity analysis. For this score, the participants filled out a food propensity questionnaire surveying daily food choices within the past 30 days. The survey and its scoring system has been described in more detail by Koponen *et al.* (3). In short, scores were rewarded based on healthy food choices, having the Nordic Nutrition Recommendations as reference. The final score, which ranged from 9 to 745, measured the total monthly consumption of healthy food choices, with higher scores rewarding healthier eating.

Prior use of antibiotics was extracted from the Drug Purchase Register and defined as a purchase of medications with an Anatomical Therapeutic Chemical (ATC) code of J01 up to 4 months before baseline. The incident infection and prevalent disease status were both defined by using the codes stored in the Hospital Discharge Register or Medication Purchase register (**Table E1**).

*Statistical analysis*

We summarized categorical variables as percentages and compared proportions with the χ^2^ test. Continuous data were presented as median with interquartile range and differences between groups were compared using the Kruskal–Wallis test. All hazard ratios were calculated per one standard deviation (SD) increase.

We quantified alpha diversity using the Shannon Diversity Index at the species level after rarefaction (vegan package). We assessed community composition (beta diversity) using the Bray-Curtis dissimilarity (phyloseq R package) and principal coordinates. We calculated Spearman correlation for taxa on defined axes to identify most strongly correlated taxa (**Figure 3**). The graph illustrating how the PCoA axes explain the variance was used to determine how many were used in the analyses (**Figure E3**).

We defined common taxa (genera and species separately) as having a compositional detection rate of at least 0.1% and a prevalence of 1%. The total relative butyrate abundance was calculated as the sum of 16 pre-defined taxa that represent the most abundant butyrate producers (**Table E2**) (4). We used the CRL-transformed microbial abundances for taxa-level analyses to correct for the compositional nature of the data.

Similar to our earlier work (5), we used elastic nets for Cox models to develop a bacterial risk score for incident pneumonia. Based on a method by Peled et al. (6), we calculated a risk score based on the effect size (weight) of the relation between common species and the risk of pneumonia in the FINRISK cohort (**Figure E2**). The relative abundance of common species were log transformed after adding a pseudocount of 2x10^–5^. Effect sizes were calculated by regularized Cox regression using cv.glmnet function from glmnet package (10-fold cross-validation, maximum number of iterations 10000), and normalized. For a sensitivity analysis, we randomly divided the cohort population into two equal groups: discovery and test halves. We first defined the risk score in the discovery half of the population, observing a hazard ratio (HR) of 1.43 (95% CI 1.29–1.59, P < 0.001) for pneumonia per 1-SD increase in the risk score. This association was consistent in the test half of the population, where the HR was 1.24 (95% CI 1.13–1.39, P < 0.001) per 1-SD increase in the risk score. Our final risk score is constructed using the entire cohort population for an optimized model. The main features of the risk score are listed in **Table E5**.

We used R (survival package) to test for Cox Proportional Hazards Assumption of the risk score model. The risk score did not have time-significant association with model residuals (p=0.52).

**Supplemental Tables**

**Table E1.** Coding of register-based diagnoses in FINRISK.

| **Diagnosis** | **Hospital discharge and death registers (ICD-10)** | **Drug reimbursement and purchase registers (ATC codes or drug reimbursement codes)** |
| --- | --- | --- |
| Pneumonia | J1[2-6,8]\|J17.0*\|J17[1-8]\|J100\|J110\|B012\|B068\|B250 |  |
| Prevalent diabetes | E1[0-4] |  |
| Prevalent cardiovascular disease | I200\|I21\|I22\|I61\|I63\|I64 |  |
| Prevalent cancer | C[0-3]\|C4[0-3]\|C4[5-9]\|C[5-9] |  |
| Prevalent hypertension | I10 | DR: 205 |
| Prevalent pulmonary disease | J43\|J44\|J45\|J46 | DR: 203  ATC: R03BA\|R03BC\|R03DC\|R03AK |
| Prevalent gastroenterological disease | K7[0-7]\|K900\|K50\|K51 | DR: 208 |

ATC, Anatomical Therapeutic Chemical Classification; ICD-10, International Classification of Diseases 10th Revision; DR, Drug reimbursement register;

**Table E2.** The 16 taxa used to define the abundance of butyrate-producing bacteria in our study.

| **Phylum** | **Order** | **Class** | **Family** | **Genus** | **Species** |
| --- | --- | --- | --- | --- | --- |
| *Bacteroidota* | *Bacteroidia* | *Bacteroidales* | *Marinifilaceae* | *Butyricimonas* |  |
| *Bacteroidota* | *Bacteroidia* | *Bacteroidales* | *Marinifilaceae* | *Odoribacter* |  |
| *Firmicutes* | *Clostridia* | *Lachnospirales* | *Lachnospiraceae* | *Agathobacter* |  |
| *Firmicutes* | *Clostridia* | *Lachnospirales* | *Lachnospiraceae* | *Anaerobutyricum* |  |
| *Firmicutes* | *Clostridia* | *Lachnospirales* | *Lachnospiraceae* | *Eubacterium* | *Ventriosum* |
| *Firmicutes* | *Clostridia* | *Lachnospirales* | *Lachnospiraceae* | *Anaerostipes* |  |
| *Firmicutes* | *Clostridia* | *Lachnospirales* | *Lachnospiraceae* | *Butyrivibrio* |  |
| *Firmicutes* | *Clostridia* | *Lachnospirales* | *Lachnospiraceae* | *Coprococcus* |  |
| *Firmicutes* | *Clostridia* | *Lachnospirales* | *Lachnospiraceae* | *Roseburia* |  |
| *Firmicutes* | *Clostridia* | *Lachnospirales* | *Lachnospiraceae* | *Shuttleworthia* |  |
| *Firmicutes* | *Clostridia* | *Clostridiales* | *Butyricicoccaceae* | *Butyricicoccus* |  |
| *Firmicutes* | *Clostridia* | *Oscillospirales* | *Ruminococcaceae* | *Faecalibacterium* |  |
| *Firmicutes* | *Clostridia* | *Oscillospirales* | *Ruminococcaceae* | *Subdoligranulum* |  |
| *Firmicutes* | *Clostridia* | *Oscillospirales* | *Oscillospiraceae* | *Flavonifractor* |  |
| *Firmicutes* | *Clostridia* | *Oscillospirales* | *Oscillospiraceae* | *Pseudoflavonifractor* |  |
| *Firmicutes* | *Clostridia* | *Oscillospirales* | *Oscillospiraceae* | *Oscillibacter* |  |

These bacteria have been recognized as the predominant promoters of intestinal butyrate synthesis (4)

**Table E3.** The unadjusted associations between genus-level taxa and incident pneumonia.

| **Predictor** | **HR** | **P-value** | **P (adjusted)** |
| --- | --- | --- | --- |
| *Eubacterium_G* | 0.802 (95% CI, 0.746-0.863) | 0.000000004 | 0.000000482 |
| *Faecalibacillus* | 0.806 (95% CI, 0.75-0.867) | 0.000000006 | 0.000000531 |
| *Anaerostipes* | 0.812 (95% CI, 0.753-0.876) | 0.000000067 | 0.000004249 |
| *Fusicatenibacter* | 0.834 (95% CI, 0.776-0.897) | 0.000000873 | 0.000036815 |
| *Eubacterium_I* | 0.838 (95% CI, 0.778-0.904) | 0.000004102 | 0.000094352 |
| *Faecalibacterium* | 0.848 (95% CI, 0.789-0.91) | 0.000005876 | 0.000114349 |
| *Anaerobutyricum* | 0.848 (95% CI, 0.788-0.912) | 0.000009606 | 0.000173590 |
| *Adlercreutzia_404257* | 0.849 (95% CI, 0.793-0.909) | 0.000002925 | 0.000075681 |
| *Scatocola* | 0.852 (95% CI, 0.789-0.92) | 0.000044502 | 0.000625504 |
| *14-2* | 0.856 (95% CI, 0.796-0.919) | 0.000021867 | 0.000345765 |
| *CAG-603* | 0.858 (95% CI, 0.8-0.92) | 0.000016591 | 0.000279841 |
| *CAG-1427* | 0.857 (95% CI, 0.794-0.924) | 0.000065752 | 0.000831767 |
| *UMGS1375* | 0.871 (95% CI, 0.807-0.939) | 0.000337057 | 0.003158347 |
| *CAG-793* | 0.876 (95% CI, 0.819-0.937) | 0.000110064 | 0.001210703 |
| *Dorea_A* | 0.877 (95% CI, 0.816-0.944) | 0.000460324 | 0.004015931 |
| *Eisenbergiella* | 0.878 (95% CI, 0.815-0.945) | 0.000571247 | 0.004817516 |
| *Lactococcus_A_346120* | 0.878 (95% CI, 0.816-0.944) | 0.000415300 | 0.003752529 |
| *Blautia_A_141781* | 0.883 (95% CI, 0.818-0.953) | 0.001335745 | 0.009655532 |
| *CAG-353* | 0.883 (95% CI, 0.815-0.958) | 0.002694724 | 0.013367946 |
| *Lactonifactor* | 0.887 (95% CI, 0.822-0.957) | 0.001905166 | 0.011341629 |
| *Limivicinus* | 0.887 (95% CI, 0.821-0.958) | 0.002171709 | 0.011944401 |
| *UMGS1071* | 0.888 (95% CI, 0.826-0.954) | 0.001246689 | 0.009276833 |
| *Marvinbryantia* | 0.888 (95% CI, 0.825-0.956) | 0.001635253 | 0.010823156 |
| *Lachnospira* | 0.888 (95% CI, 0.825-0.956) | 0.001687027 | 0.010823156 |
| *Acetatifactor* | 0.889 (95% CI, 0.825-0.958) | 0.001972457 | 0.011341629 |
| *AM51-8* | 0.89 (95% CI, 0.827-0.957) | 0.001690415 | 0.010823156 |
| *Schaedlerella* | 0.889 (95% CI, 0.824-0.959) | 0.002438136 | 0.012851010 |
| *Bacteroides_F* | 0.891 (95% CI, 0.827-0.959) | 0.002048887 | 0.011519295 |
| *Soehngenia_A_223990* | 0.891 (95% CI, 0.828-0.96) | 0.002339755 | 0.012594852 |
| *Barnesiella* | 0.894 (95% CI, 0.831-0.962) | 0.002670660 | 0.013367946 |
| *Gemmiger_A_73129* | 0.895 (95% CI, 0.833-0.962) | 0.002504906 | 0.012933494 |
| *Bifidobacterium_388775* | 0.898 (95% CI, 0.836-0.965) | 0.003466154 | 0.016864171 |
| *CAG-267* | 0.898 (95% CI, 0.83-0.971) | 0.007015655 | 0.030184206 |
| *Mediterraneibacter_A_155590* | 0.899 (95% CI, 0.835-0.967) | 0.004207112 | 0.020083005 |
| *CAG-95* | 0.9 (95% CI, 0.836-0.969) | 0.005095518 | 0.023439384 |
| *UBA3402* | 0.901 (95% CI, 0.836-0.97) | 0.005609683 | 0.025343746 |
| *CAG-45* | 0.903 (95% CI, 0.838-0.973) | 0.007039005 | 0.030184206 |
| *Haemophilus_D_735815* | 0.904 (95% CI, 0.84-0.973) | 0.006924416 | 0.030184206 |
| *Ventrimonas* | 0.904 (95% CI, 0.839-0.975) | 0.008905046 | 0.036934043 |
| *Agathobacter_164117* | 0.912 (95% CI, 0.849-0.98) | 0.012288907 | 0.049350691 |
| *Absicoccus* | 1.104 (95% CI, 1.026-1.188) | 0.007782949 | 0.032818103 |
| *Clostridium_T* | 1.105 (95% CI, 1.025-1.191) | 0.009068708 | 0.037006179 |
| *Lacrimispora* | 1.115 (95% CI, 1.034-1.203) | 0.004766197 | 0.022330515 |
| *Faecalimonas* | 1.119 (95% CI, 1.044-1.199) | 0.001459235 | 0.010255177 |
| *Catenibacterium* | 1.119 (95% CI, 1.043-1.201) | 0.001711171 | 0.010823156 |
| *Holdemanella* | 1.122 (95% CI, 1.044-1.205) | 0.001814167 | 0.011194739 |
| *Phascolarctobacterium_A* | 1.123 (95% CI, 1.043-1.209) | 0.001970681 | 0.011341629 |
| *Clostridium_AQ* | 1.128 (95% CI, 1.05-1.212) | 0.000991024 | 0.007597850 |
| *CAG-217* | 1.135 (95% CI, 1.055-1.222) | 0.000696388 | 0.005505817 |
| *Ligilactobacillus* | 1.138 (95% CI, 1.062-1.219) | 0.000251859 | 0.002548815 |
| *Megasphaera_A_38685* | 1.143 (95% CI, 1.065-1.225) | 0.000185709 | 0.001957682 |
| *Paratractidigestivibacter* | 1.142 (95% CI, 1.058-1.233) | 0.000687258 | 0.005505817 |
| *Escherichia_710834* | 1.15 (95% CI, 1.066-1.24) | 0.000289075 | 0.002812919 |
| *Citrobacter_A_692098* | 1.164 (95% CI, 1.081-1.254) | 0.000061210 | 0.000815060 |
| *Enterobacter_B_713587* | 1.165 (95% CI, 1.08-1.257) | 0.000086268 | 0.001028887 |
| *Ellagibacter* | 1.165 (95% CI, 1.079-1.257) | 0.000089468 | 0.001028887 |
| *Ruminococcus_B* | 1.171 (95% CI, 1.094-1.253) | 0.000005832 | 0.000114349 |
| *Salmonella_692099* | 1.172 (95% CI, 1.086-1.265) | 0.000043339 | 0.000625504 |
| *Fimenecus* | 1.182 (95% CI, 1.102-1.268) | 0.000002991 | 0.000075681 |
| *VUNA01* | 1.19 (95% CI, 1.108-1.277) | 0.000001499 | 0.000049295 |
| *Slackia_A* | 1.203 (95% CI, 1.115-1.297) | 0.000001559 | 0.000049295 |
| *Klebsiella_724518* | 1.213 (95% CI, 1.125-1.309) | 0.000000616 | 0.000031146 |
| *Limosilactobacillus* | 1.261 (95% CI, 1.169-1.361) | 0.000000002 | 0.000000482 |

Abbreviations: HR = hazard ratio, CI = confidence interval

**Table E4:** The unadjusted associations between bacterial species and incident pneumonia.

| **Predictor** | **HR (95% CI)** | **P-value** | **P (adjusted)** |
| --- | --- | --- | --- |
| *Eubacterium_G ventriosum* | 0.792 (95% CI, 0.734-0.855) | 0.000000002 | 0.000000698 |
| *Faecalibacillus intestinalis* | 0.807 (95% CI, 0.751-0.868) | 0.000000007 | 0.000001394 |
| *Anaerostipes hadrus* | 0.811 (95% CI, 0.753-0.873) | 0.000000024 | 0.000003126 |
| *CAG-1427 sp000435675* | 0.813 (95% CI, 0.751-0.88) | 0.000000300 | 0.000026613 |
| *Blautia_A_141781 massiliensis* | 0.82 (95% CI, 0.76-0.886) | 0.000000420 | 0.000026856 |
| *Mediterraneibacter_A_155507 faecis* | 0.83 (95% CI, 0.77-0.895) | 0.000001308 | 0.000049621 |
| *Fusicatenibacter saccharivorans* | 0.836 (95% CI, 0.777-0.898) | 0.000001136 | 0.000049621 |
| *Eubacterium_I ramulus* | 0.836 (95% CI, 0.775-0.901) | 0.000002874 | 0.000084901 |
| *Anaerobutyricum hallii* | 0.84 (95% CI, 0.782-0.902) | 0.000001665 | 0.000053273 |
| *Adlercreutzia equolifaciens* | 0.844 (95% CI, 0.789-0.903) | 0.000000758 | 0.000041562 |
| *Faecalibacterium prausnitzii_C_71351* | 0.847 (95% CI, 0.789-0.909) | 0.000003993 | 0.000104708 |
| *Schaedlerella sp900066545* | 0.848 (95% CI, 0.787-0.914) | 0.000017914 | 0.000374848 |
| *Lachnospira sp000436535* | 0.85 (95% CI, 0.788-0.916) | 0.000023601 | 0.000431563 |
| *Scatocola faecipullorum* | 0.855 (95% CI, 0.792-0.923) | 0.000062721 | 0.000928890 |
| *Butyribacter intestini* | 0.858 (95% CI, 0.795-0.925) | 0.000062894 | 0.000928890 |
| *CAG-603 sp900066105* | 0.859 (95% CI, 0.801-0.921) | 0.000021452 | 0.000411879 |
| *Lactococcus_A_346120 lactis_344179* | 0.869 (95% CI, 0.808-0.934) | 0.000146227 | 0.001871701 |
| *Bifidobacterium bifidum* | 0.87 (95% CI, 0.806-0.938) | 0.000321754 | 0.003339289 |
| *Blautia_A_141781 sp900066505* | 0.872 (95% CI, 0.81-0.939) | 0.000266816 | 0.002972806 |
| *UMGS1375 sp900066615* | 0.872 (95% CI, 0.809-0.941) | 0.000401297 | 0.003852451 |
| *Lactobacillus delbrueckii* | 0.872 (95% CI, 0.806-0.943) | 0.000588410 | 0.005379746 |
| *Agathobacter rectalis* | 0.875 (95% CI, 0.814-0.94) | 0.000283566 | 0.003024706 |
| *CAG-793 sp000433915* | 0.877 (95% CI, 0.82-0.938) | 0.000131883 | 0.001746309 |
| *51-20 sp001917175* | 0.881 (95% CI, 0.814-0.954) | 0.001719447 | 0.011383924 |
| *Erysipelatoclostridium spiroforme* | 0.883 (95% CI, 0.822-0.949) | 0.000690521 | 0.005892447 |
| *Bifidobacterium longum* | 0.883 (95% CI, 0.824-0.946) | 0.000412819 | 0.003866401 |
| *Bacteroides_H thetaiotaomicron* | 0.884 (95% CI, 0.822-0.949) | 0.000723448 | 0.006039215 |
| *Dorea_A longicatena* | 0.885 (95% CI, 0.823-0.952) | 0.000965137 | 0.007412252 |
| *Eubacterium_G sp000434315* | 0.885 (95% CI, 0.819-0.956) | 0.001847392 | 0.012023703 |
| *CAG-353 sp900066885* | 0.885 (95% CI, 0.816-0.96) | 0.003063017 | 0.017046355 |
| *Marvinbryantia sp900066075* | 0.886 (95% CI, 0.823-0.954) | 0.001259848 | 0.009399040 |
| *UMGS1071 sp900548305* | 0.889 (95% CI, 0.827-0.956) | 0.001420172 | 0.010139769 |
| *Bacteroides_H xylanisolvens* | 0.889 (95% CI, 0.826-0.956) | 0.001515766 | 0.010582805 |
| *Blautia_A_141781 obeum* | 0.889 (95% CI, 0.825-0.959) | 0.002189012 | 0.013780012 |
| *Acetatifactor sp900066565* | 0.889 (95% CI, 0.826-0.958) | 0.001915353 | 0.012258261 |
| *Lactonifactor longoviformis* | 0.891 (95% CI, 0.826-0.961) | 0.002820664 | 0.016166195 |
| *Limivicinus sp002320035* | 0.89 (95% CI, 0.824-0.961) | 0.002992032 | 0.016896182 |
| *Lachnospira sp000437735* | 0.89 (95% CI, 0.824-0.962) | 0.003149804 | 0.017278924 |
| *Enterocloster sp000431375* | 0.891 (95% CI, 0.824-0.963) | 0.003826651 | 0.019857217 |
| *AM51-8 sp003478275* | 0.892 (95% CI, 0.829-0.96) | 0.002330737 | 0.014435535 |
| *Bacteroides_F pectinophilus* | 0.893 (95% CI, 0.829-0.961) | 0.002566457 | 0.015161836 |
| *Eubacterium_G sp000435815* | 0.892 (95% CI, 0.826-0.963) | 0.003488410 | 0.018604853 |
| *Bacteroides_H reticulotermitis* | 0.893 (95% CI, 0.836-0.954) | 0.000783967 | 0.006271737 |
| *Barnesiella intestinihominis* | 0.893 (95% CI, 0.83-0.961) | 0.002383231 | 0.014526360 |
| *Odoribacter splanchnicus* | 0.898 (95% CI, 0.837-0.963) | 0.002514475 | 0.015086852 |
| *Bifidobacterium angulatum* | 0.898 (95% CI, 0.837-0.963) | 0.002682052 | 0.015604669 |
| *Agathobaculum butyriciproducens* | 0.898 (95% CI, 0.835-0.966) | 0.003643014 | 0.019163253 |
| *Erysipelatoclostridium saccharogumia* | 0.899 (95% CI, 0.833-0.97) | 0.006213364 | 0.028403951 |
| *CAG-267 sp001917135* | 0.899 (95% CI, 0.831-0.972) | 0.007798999 | 0.034031998 |
| *Streptococcus thermophilus* | 0.901 (95% CI, 0.837-0.969) | 0.005006306 | 0.024334447 |
| *Mediterraneibacter_A_155590 butyricigenes* | 0.901 (95% CI, 0.837-0.97) | 0.005574626 | 0.026427858 |
| *CAG-95 sp900066375* | 0.902 (95% CI, 0.838-0.971) | 0.006130809 | 0.028364227 |
| *Dorea_A formicigenerans* | 0.904 (95% CI, 0.839-0.973) | 0.007209283 | 0.032568996 |
| *UBA3402 sp003478355* | 0.904 (95% CI, 0.839-0.973) | 0.007559713 | 0.033754997 |
| *Blautia_A_141781 wexlerae* | 0.904 (95% CI, 0.839-0.975) | 0.008597884 | 0.035310177 |
| *Bifidobacterium breve* | 0.905 (95% CI, 0.845-0.969) | 0.004313123 | 0.021509602 |
| *Haemophilus_D_735815 parainfluenzae_K_735050* | 0.905 (95% CI, 0.841-0.975) | 0.008304593 | 0.035043556 |
| *CAG-45 sp000438375* | 0.905 (95% CI, 0.84-0.975) | 0.008671035 | 0.035310177 |
| *Coprobacter fastidiosus* | 0.905 (95% CI, 0.844-0.972) | 0.005852442 | 0.027406559 |
| *Gemmiger_A_73129 qucibialis* | 0.906 (95% CI, 0.842-0.975) | 0.008157778 | 0.034806518 |
| *CAG-313 sp000433035* | 0.906 (95% CI, 0.84-0.977) | 0.009991912 | 0.039555610 |
| *Bacteroides_H oleiciplenus* | 0.909 (95% CI, 0.846-0.976) | 0.008735591 | 0.035310177 |
| *Bifidobacterium adolescentis* | 0.908 (95% CI, 0.844-0.977) | 0.009698324 | 0.038793296 |
| *Emergencia timonensis* | 0.908 (95% CI, 0.843-0.979) | 0.011473209 | 0.044057122 |
| *Bacteroides_H salyersiae* | 0.91 (95% CI, 0.848-0.976) | 0.008640824 | 0.035310177 |
| *Lachnoclostridium_B sp900066555* | 0.909 (95% CI, 0.844-0.978) | 0.011008003 | 0.042697708 |
| *Eisenbergiella tayi* | 1.099 (95% CI, 1.023-1.181) | 0.010302329 | 0.040368308 |
| *Faecalimonas phoceensis* | 1.1 (95% CI, 1.026-1.18) | 0.007741610 | 0.034031998 |
| *Prevotella copri* | 1.101 (95% CI, 1.025-1.182) | 0.008113202 | 0.034806518 |
| *Enterocloster bolteae* | 1.107 (95% CI, 1.032-1.187) | 0.004285504 | 0.021509602 |
| *Catenibacterium mitsuokai* | 1.111 (95% CI, 1.034-1.194) | 0.004124400 | 0.021116929 |
| *Clostridium_T paraputrificum_208099* | 1.112 (95% CI, 1.032-1.198) | 0.005573149 | 0.026427858 |
| *Duodenibacillus sp900542805* | 1.113 (95% CI, 1.037-1.195) | 0.003219838 | 0.017414333 |
| *Limosilactobacillus mucosae* | 1.113 (95% CI, 1.034-1.198) | 0.004439139 | 0.021854224 |
| *Cryptobacteroides sp900316045* | 1.116 (95% CI, 1.042-1.195) | 0.001715621 | 0.011383924 |
| *Faecalimonas umbilicata* | 1.117 (95% CI, 1.044-1.195) | 0.001272787 | 0.009399040 |
| *Holdemanella biformis* | 1.123 (95% CI, 1.045-1.207) | 0.001586359 | 0.010877890 |
| *Clostridium_AQ innocuum* | 1.129 (95% CI, 1.051-1.213) | 0.000887662 | 0.006956375 |
| *Collinsella bouchesdurhonensis* | 1.132 (95% CI, 1.049-1.222) | 0.001425905 | 0.010139769 |
| *CAG-217 sp000436335* | 1.136 (95% CI, 1.056-1.222) | 0.000657561 | 0.005738714 |
| *Shigella boydii* | 1.142 (95% CI, 1.057-1.233) | 0.000755332 | 0.006171226 |
| *Paratractidigestivibacter faecalis* | 1.144 (95% CI, 1.059-1.235) | 0.000606626 | 0.005417308 |
| *Megasphaera_A_38685 elsdenii* | 1.145 (95% CI, 1.069-1.226) | 0.000111845 | 0.001533874 |
| *Citrobacter_A_692098 werkmanii* | 1.148 (95% CI, 1.066-1.235) | 0.000247922 | 0.002972806 |
| *Escherichia ruysiae* | 1.149 (95% CI, 1.064-1.241) | 0.000399442 | 0.003852451 |
| *Escherichia marmotae* | 1.151 (95% CI, 1.065-1.243) | 0.000363638 | 0.003674655 |
| *Shigella sonnei* | 1.153 (95% CI, 1.068-1.245) | 0.000270959 | 0.002972806 |
| *Escherichia coli* | 1.155 (95% CI, 1.069-1.247) | 0.000253238 | 0.002972806 |
| *Escherichia albertii* | 1.155 (95% CI, 1.069-1.247) | 0.000261691 | 0.002972806 |
| *Ellagibacter isourolithinifaciens* | 1.165 (95% CI, 1.08-1.258) | 0.000084582 | 0.001202944 |
| *Klebsiella aerogenes_724207* | 1.167 (95% CI, 1.087-1.252) | 0.000018547 | 0.000374848 |
| *Klebsiella michiganensis* | 1.167 (95% CI, 1.085-1.255) | 0.000032202 | 0.000562068 |
| *Ruminococcus_B gnavus* | 1.172 (95% CI, 1.095-1.254) | 0.000005030 | 0.000120709 |
| *Salmonella enterica_690914* | 1.172 (95% CI, 1.087-1.265) | 0.000041265 | 0.000660243 |
| *Phascolarctobacterium_A succinatutens* | 1.178 (95% CI, 1.099-1.263) | 0.000004090 | 0.000104708 |
| *Slackia_A isoflavoniconvertens* | 1.179 (95% CI, 1.091-1.274) | 0.000033975 | 0.000567239 |
| *Enterobacter_B_713587 kobei_713570* | 1.182 (95% CI, 1.095-1.275) | 0.000015996 | 0.000361318 |
| *Fimenecus sp000432435* | 1.188 (95% CI, 1.108-1.274) | 0.000001421 | 0.000049621 |
| *VUNA01 sp002299625* | 1.191 (95% CI, 1.11-1.278) | 0.000001295 | 0.000049621 |
| *Klebsiella pneumoniae_718977* | 1.213 (95% CI, 1.126-1.306) | 0.000000347 | 0.000026613 |

Abbreviations: HR = hazard ratio, CI = confidence interval

**Table E5.** Results from a sensitivity analysis which included Healthy Food Choices score as a covariate in the Cox proportional hazards model. Results are reported for taxa that were significantly associated with incident pneumonia in the main analysis.

| Taxa | HR | 95% CI | *P* | FDR |
| --- | --- | --- | --- | --- |
| Genus |  |  |  |  |
| *Eubacterium_G* | 0.87 | 0.80–0.94 | 5.0 x 10^-4^ | 0.08 |
| *Bacteroides_F* | 0.88 | 0.81–0.96 | 2.6 x 10^-3^ | 0.08 |
| *CAG_603* | 0.89 | 0.83–0.97 | 4.8 x 10^-3^ | 0.11 |
| *Clostridium_AQ* | 1.13 | 1.05–1.22 | 1.2 x 10^-3^ | 0.08 |
| *Slackia_A* | 1.14 | 1.05–1.23 | 2.1 x 10^-3^ | 0.08 |
| Species |  |  |  |  |
| *Eubacterium_G ventriosum* | 0.85 | 0.78–0.92 | 7.7 x 10^-5^ | 0.03 |
| *Agathobaculum butyriciproducens* | 0.89 | 0.82–0.96 | 4.2 x 10^-3^ | 0.11 |
| *CAG-1427 sp000435675* | 0.87 | 0.80–0.94 | 1.1 x 10^-3^ | 0.11 |
| *Bacteroides_F pectinophilus* | 0.88 | 0.81–0.96 | 2.5 x 10^-3^ | 0.11 |
| *Butyribacter intestini* | 0.88 | 0.81–0.96 | 3.6 x 10^-3^ | 0.11 |
| *Eubacterium_I ramulus* | 0.89 | 0.82–0.97 | 5.3 x 10^-3^ | 0.11 |
| *CAG-603 sp900066105* | 0.89 | 0.83–0.97 | 4.6 x 10^-3^ | 0.11 |
| *Clostridium_AQ innocuum* | 1.13 | 1.05–1.22 | 1.4 x 10^-3^ | 0.11 |

Hazard ratios are reported per 1 SD increment. Abbreviations: HR = Hazard ratio, CI = Confident interval, FDR = False discovery rate corrected *P.*

**Table E6:** The main contributors of the microbial risk score for pneumonia.

| **Species** | **Coefficient** |
| --- | --- |
| *VUNA01 sp002299625* | 0.070507830 |
| *Ruminococcus_B gnavus* | 0.059828703 |
| *Klebsiella pneumoniae_718977* | 0.049333889 |
| *Slackia_A isoflavoniconvertens* | 0.046058460 |
| *Fimenecus sp000432435* | 0.022389597 |
| *Phascolarctobacterium_A succinatutens* | 0.013629688 |
| *Erysipelatoclostridium ramosum* | 0.004648576 |
| *Klebsiella michiganensis* | 0.001276610 |
| *Clostridium_AQ innocuum* | 0.000896849 |
| *Lactobacillus delbrueckii* | -0.000537101 |
| *Bifidobacterium angulatum* | -0.001862971 |
| *Adlercreutzia equolifaciens* | -0.004803255 |
| *CAG-1427 sp000435675* | -0.009417042 |
| *Anaerostipes hadrus* | -0.017222753 |
| *Blautia_A_141781 massiliensis* | -0.020601884 |
| *Mediterraneibacter_A_155507 faecis* | -0.021755706 |
| *Lactococcus_A_346120 lactis_344179* | -0.025509233 |
| *Faecalibacillus intestinalis* | -0.029353220 |
| *Eubacterium_G ventriosum* | -0.066204714 |

**Supplemental Figures**

**Figure E1.** The contribution of common bacterial taxa to the risk score.


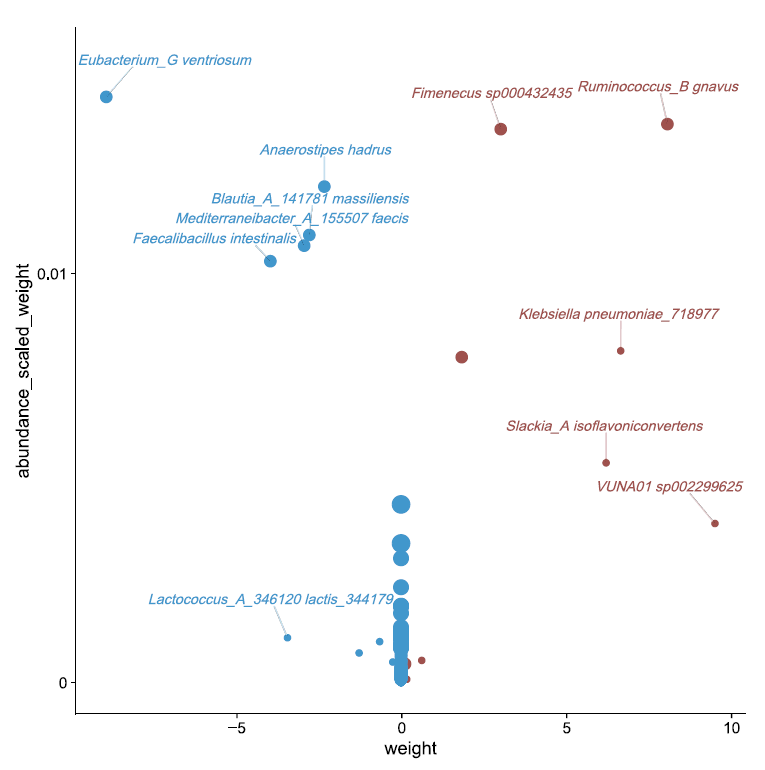


Every dot symbolizes a different bacterial genus, with the size of the dots correlating to the average relative abundance. The effect size (weight) defined by regularized Cox regression is displayed on the horizontal axis. The total contribution to the risk score is depicted on the vertical axis, calculated by multiplying weight with abundance.

**Figure E2.** The microbiota-based risk score association with pneumonia incidence.


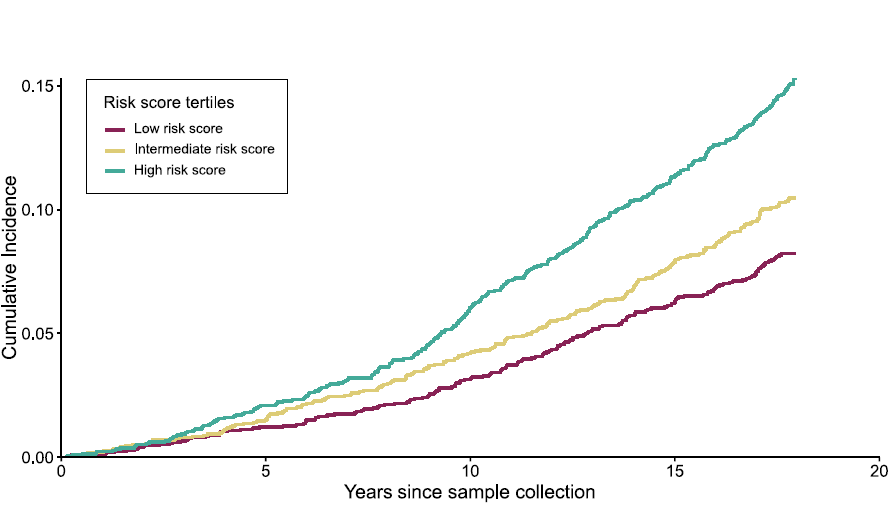


The cumulative incidence of pneumonia in the study cohort, divided into tertiles according to the calculated risk score.

**Figure E3.** Variance explained by the PCoA axes.


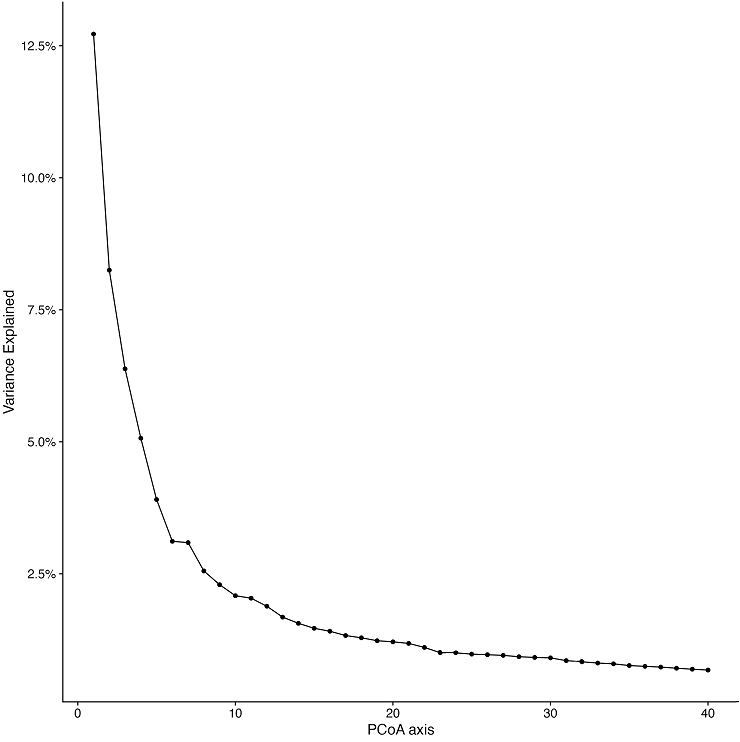


The graph illustrates the extent to which specific PCoA axes explain the overall variance in the dataset. Abbreviations: PCoA = Principal coordinate analysis.

**References for Online Supplement**

1. Gissler M, Haukka J. Finnish health and social welfare registers in epidemiological research. *Norsk Epidemiologi* 2004;14:113–120.

2. Sund R. Quality of the Finnish Hospital Discharge Register: a systematic review. *Scand J Public Health* 2012;40:505–515.

3. Koponen KK, Salosensaari A, Ruuskanen MO, Havulinna AS, Männistö S, Jousilahti P, *et al.* Associations of healthy food choices with gut microbiota profiles. *The American Journal of Clinical Nutrition* 2021;114:605.

4. Vital M, Karch A, Pieper DH. Colonic Butyrate-Producing Communities in Humans: an Overview Using Omics Data. *mSystems* 2017;2:e00130-17.

5. Kullberg RFJ, Wikki I, Haak BW, Kauko A, Galenkamp H, Peters-Sengers H, *et al.* Association between butyrate-producing gut bacteria and the risk of infectious disease hospitalisation: results from two observational, population-based microbiome studies. *The Lancet Microbe* 2024;5:100864.

6. Peled JU, Gomes ALC, Devlin SM, Littmann ER, Taur Y, Sung AD, *et al.* Microbiota as Predictor of Mortality in Allogeneic Hematopoietic-Cell Transplantation. *N Engl J Med* 2020;382:822–834.
